# Molecular Characterization of Bacterial Isolates from Soil Samples and Evaluation of their Antibacterial Potential against MDRS

**DOI:** 10.3390/molecules27196281

**Published:** 2022-09-23

**Authors:** Shahida Sadiqi, Muhammad Hamza, Farooq Ali, Sadia Alam, Qismat Shakeela, Shehzad Ahmed, Asma Ayaz, Sajid Ali, Saddam Saqib, Fazal Ullah, Wajid Zaman

**Affiliations:** 1 Department of Microbiology, Faculty of Biological and Health Sciences, Hazara University Mansehra, Mansehra 21300, KP, Pakistan; 2 Department of Microbiology, Faculty of Biological and Biomedical Sciences, University of Haripur, Haripur 21120, KP, Pakistan; 3 Department of Microbiology, Faculty of Health Sciences, University of Science and Technology, Abbottabad 22020, KP, Pakistan; 4 State Key Laboratory of Biocatalysis and Enzyme Engineering, School of Life Sciences, Hubei University, Wuhan 430062, China; 5 Department of Horticulture and Life Science, Yeungnam University, Gyeongsan 38541, Korea; 6 State Key Laboratory of Systematic and Evolutionary Botany, Institute of Botany, Chinese Academy of Sciences, Beijing 100093, China; 7 University of Chinese Academy of Sciences, Beijing 100049, China; 8 State Key Laboratory of Grassland Agro-Ecosystems, School of Life Sciences, Lanzhou University, Lanzhou 730000, China; 9 Department of Life Sciences, Yeungnam University, Gyeongsan 38541, Korea

**Keywords:** multidrug-resistant strains, antimicrobial activity, secondary metabolites, *Bacillus*, soil

## Abstract

Some soil microbes, with their diverse inhabitance, biologically active metabolites, and endospore formation, gave them characteristic predominance and recognition among other microbial communities. The present study collected ten soil samples from green land, agricultural and marshy soil sites of Khyber Pakhtunkhwa, Pakistan. After culturing on described media, the bacterial isolates were identified through phenotypic, biochemical and phylogenetic analysis. Our phylogenetic analysis revealed three bacterial isolates, A6S7, A1S6, and A1S10, showing 99% nucleotides sequence similarity with *Brevibacillus formosus*, *Bacillus Subtilis* and *Paenibacillus dendritiformis.* The crude extract was prepared from bacterial isolates to assess the anti-bacterial potential against various targeted multidrug-resistant strains (MDRS), including *Acinetobacter baumannii* (ATCC 19606), Methicillin-resistant *Staphylococcus aureus* (MRSA) (BAA-1683), *Klebsiella pneumoniae* (ATCC 13883)*, Pseudomonas aeruginosa* (BAA-2108)*, Staphylococcus aureus* (ATCC 292013)*, Escherichia coli* (ATCC25922) and *Salmonella typhi* (ATCC 14028). Our analysis revealed that all bacterial extracts possess activity against Gram-negative and Gram-positive bacteria at a concentration of 5 mg/mL, efficiently restricting the growth of *E. coli* compared with positive control ciprofloxacin. The study concluded that the identified species have the potential to produce antimicrobial compounds which can be used to control different microbial infections, especially MDRS. Moreover, the analysis of the bacterial extracts through GC-MS indicated the presence of different antimicrobial compounds such as propanoic acid, oxalic acid, phenol and hexadecanoic acid.

## 1. Introduction

Antibiotic resistance in bacterial strains is taking root throughout the population and poses a serious public health challenge, which greatly demands pursuing new antibiotics or investigating new antimicrobial compounds [1]. During the last few decades, much work has been documented, including the research on the production of new antibiotics from diverse strains of microorganisms and plants [2,3,4,5]. These microbial species and their population size depend on environmental factors such as soil texture, nutrient availability, moisture, and flora-covered soil [6]. Bacteria produce and use antibiotics in their natural habitats as protective substances to destroy the invasion of other bacterial species. The function of these antibiotics is not only protective but they also play a vital role as signalling molecules to communicate among the cells in the bacterial population [7,8].

It is presumed from the past that natural products play a vital role in antibiotic discovery and their development [9]. There is a dire need to explore novel antimicrobial compounds with significant potential to kill or control a wide range of microorganisms. Antibiotics are one of the essential pillars of present-time medications. Still, unfortunately, the commonly used antibiotics lose their efficacy against several pathogenic strains and there is demand to replace old antibiotics with new ones [10,11]. Microorganisms, having the ability to produce bioactive secondary metabolites, show distinct structures and biological activities. Some species of soil microflora also produce these bioactive metabolites, which are used as antibiotics [6]. Several other significant types of research have also been reported to isolate bacteria from the soil with novel antimicrobial agents [12,13].

Public health professionals recently had great difficulty dealing with multidrug-resistant bacterial pathogens; therefore, MDR infections are more dangerous than non-resistant bacterial pathogens. Particularly, the frequency of resistance developed in bacterial pathogens acts as a secondary infection in various life-threatening conditions such as cancer, surgical procedures, transplantation, etc., and influences the treatment impact of modern medications [14,15]. As the development of MDR strains is quite rapid, it is obvious that very limited therapeutic agents are available to treat these pathogens effectively [16,17]. This study focused on isolating potential bacterial species from the soil and preparing a crude extract from isolated bacterial strains to assess antimicrobial activities against most common human pathogens. In addition, GC-MS was performed to determine bioactive compounds from the crude extract. This study will facilitate the development of novel antibiotics against MDR bacterial strains.

## 2. Materials and Methods

### 2.1. Sample Collection and Processing

While conducting the present study, n = 10 samples were collected from different regions of Khyber Pakhtunkhwa, including Haripur, Swat, and Charsadda. Approximately 4 gm of each soil sample was collected in a sterile polythene bag using a sterile spatula. The collected samples were immediately transferred to the laboratory in ice baskets for bacteriological investigation. The pH and moisture content of each sample was also determined. Samples were processed and inoculated using suitable culture media, including nutrient agar, GYM medium, and Tryptic Soy broth with 5% yeast extract for obtaining bacterial isolates. Before inoculation, each soil sample was serially diluted in sterile normal saline and homogeneously mixed by vertexing. The inoculum (100 µL from each dilution) was spread on culture media and was allowed to incubate overnight at 37 °C. After incubation, the fresh culture was subjected to morphological identification. Slightly raised, flat, white, and cream-colored colonies were selected for further identification. Gram staining and different biochemical tests were performed for further identification. The biochemical tests included indole, methyl red, Voges–Proskauer, citrate utilization, catalase, oxidase, cellulose test, triple sugar iron, nitrate reduction, gelatin, and casein hydrolysis test.

### 2.2. Secondary Metabolites Production

The sterilized nutrient broth (150 mL) was inoculated with fresh bacterial culture in the flask and was incubated at 34 °C for seven days at 150 rpm in a shaking incubator. The cultivated bacterial strains secreted the secondary metabolites in the surrounding culture medium. After incubation, the crude broth was filtered using filter paper and was extracted with ethyl acetate using a separate funnel. The extracts were allowed to dry in a rotary evaporator. They were then stored for further processing at 4 °C.

### 2.3. Testing Bacterial Strains

Different testing bacterial strains of American-type collection centre (ATCC) culture were used as marker strains in the current study, including *Acinetobacter baumannii* (ATCC 19606), Methicillin-resistant *Staphylococcus aureus* (BAA-1683), *Klebsiella pneumoniae* (ATCC 13883), *Pseudomonas aeruginosa* (BAA-2108)*, Staphylococcus aureus* (ATCC 292013)*, Escherichia coli* (ATCC25922)*, Salmonella typhimurium* (ATCC 14028).

### 2.4. Antibacterial Activity

The antibacterial activity of isolated strains was determined against pathogenic bacteria following the well-diffusion method. The crude ethyl acetates extract dimethyl sulfoxide (DMSO) (5 mg/mL) was poured into a labelled well of MHA plate and pre-inoculated with pathogenic marker strain. The culture plates were incubated for 24 h at 37 °C to observe the antibacterial activity of each bacterial isolate by the previously described method [18]. Ciprofloxacin solution was used as a positive control with the concentration by dissolving 1 gm of ciprofloxacin per ml of DMSO, while pure DMSO was used as a negative control.

Furthermore, the broth microdilution method determined each potent bacterial extract’s minimum inhibitory concentration (MIC) [19]. Each extract’s minimum inhibitory concentration (MIC) was carried out in triplicate to measure the effective concentration against each tested pathogen. After the test application, the microtiter plate was incubated, the optical density of each isolate was incubated overnight, and each isolate’s optical density was measured at a wavelength of 600 nm using a microtiter plate reader. The minimum extract concentration able to inhibit the growth of marker/test bacteria was considered the MIC of that isolate.

### 2.5. Antifungal Activity

Using the agar dilution method, the antifungal activity of isolated bacterial isolates was checked against the two-marker fungal pathogenic strains, including *Aspergillus niger* and *Aspergillus flavus*. Seven-day-old, pathogenic marker strains of fungi were inoculated in labelled tubes containing defined crude bacterial extract 3 mg/mL. They were allowed to incubate for seven days at 27 °C in a cool incubator [20]. The growth was examined carefully and was calculated using the following formula:

$$\mathrm{Inhibition}\;\mathrm{of}\;\mathrm{fungal}\;\mathrm{growth}=\frac{\mathrm{Ig}c-\mathrm{Ig}t}{\mathrm{Ig}c\;}\;\times \;100$$

where Igc is the linear growth of the control sample while Igt is the linear growth of test sample.

### 2.6. Gas Chromatography-Mass Spectrum Analysis of Metabolites

Using Thermo Scientific GC Focus Series DSQ, bacterial secondary metabolites were analyzed by GC-MS analysis. Helium gas was used as a carrier gas at a constant flow rate of 1 mL per minute with an infection volume of 1 µL. The temperature of the injector and the hot oven was maintained at 250 °C and 110 °C, respectively, with the increase rate of 10 °C per minute up to 200 °C, then 5 °C per minute up to 280 °C and closing after 9 min at the temperature 280 °C. Peaks of varied compounds were eluted from the GC column, and their retention time was recorded. Data were matched with mass spectra of the compounds, and the database searched for similar compounds with the same molecular mass and retention time. Previously studied natural compounds reported for bioactivities were also studied, and the activities of bacterial extracts with their components in the current research were comparably correlated.

### 2.7. Molecular Characterization of Bacterial Isolates

Molecular characterization of isolated bacterial strains was based on 16S rRNA conserved gene sequences using universal bacterial primers. The targeted gene sequence was amplified using the conventional PCR method, and the size of the amplified fragments was confirmed by running the final product by 1% gel electrophoresis. The amplified samples and appropriate sequencing fragments were sent for sequencing, and the retrieved nucleotide sequences were phylogenetically studied using MEGA software (MEGA-11). Bacterial isolates were further verified/classified at the species level by BLAST search using GenBank NCBI (National Center for Biotechnology Information). To permit public access to these probiotic strains, 16S rRNA gene sequences were submitted to the GenBank database (www.ncbi.nlm.nih.gov/projects/genome/clone/) with accession numbers MT256113, MT255013, and MT256091.

## 3. Results

### 3.1. Isolation and Identification

A total of n = 10 strains of bacteria were isolated and identified based on colonial morphology, microscopy, biochemical characteristics, and sugar fermentation. Among all, Gram-positive, rod-shaped, mycelial, and spore-forming bacterial strains were selected for further confirmatory tests. The molecular analysis further validated the bacterial strains (A6S7, A1S6, A1S10) as *Brevibacillus formosus* A6S7, *Bacillus Subtilis* A1S6, *Paenibacillus dendritiformis* A1S10.

### 3.2. Morphological and Biochemical Characterization

The morphology of each colony by different bacterial isolates showed regular, irregular, slightly raised, flat, white, and cream-colored colonies. By motility test, bacterial isolates were motile and possessed terminal and subterminal spores (Table 1).

The biochemical characterizations of bacterial isolates are mentioned in (Table 2) and verified these isolates as *Bacillus subtilis*, *Brevibacillus formosus,* and *Paenibacillus dendritiformis*.

### 3.3. Carbohydrate Fermentations

The isolates showed a color change from pink to yellow, indicating that the production of fermented sugars such as glucose, sucrose, lactose, arabinose, starch, glycogen, maltose, and mannitol of a gas bubble in the Durham’s tube (Table 3).

### 3.4. Antibacterial Potency of Crude Extracts of Bacterial Isolates

**Figure 1:**
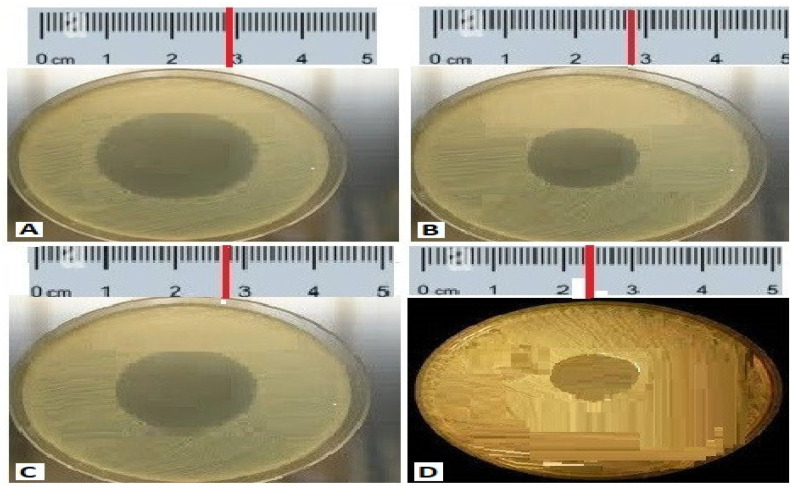
The antibacterial activity of compounds isolated from the isolated code A1 S10 *Paenibacillus* against MDR strains (**A**) *S. aureus* 28.22 mm, (**B**) *P. aeruginosa* 28 mm, (**C**) *K. Pneumoniae* 27 mm, (**D**) *Acinetobacter* *baumannii* 21 mm.

The in-vitro antibacterial potency of crude extracts of bacterial isolates against the MDR strains of both Gram-positive and Gram-negative bacteria was obtained either by the absence or presence of inhibition zones (IZ). The crude extract exhibited an inhibitory effect against the 12 MDR strains with variable diameters of inhibition zones ranging from 7 mm to 28 mm. The bacterial extracts exhibited antibacterial potency against almost all Gram-positive and Gram-negative tested strains of MDR bacteria, but with few exceptions. The best activity was shown by *Paenibacillus dendritiformis* (A1S10) against *S. aureus* with an IZ of 28.22 mm in diameter, followed by *P. aeruginosa*, *K. pneumoniae,* and *Acinetobacter* *baumannii* was 28 mm, 27 mm and 21 mm respectively. Similarly, *Brevibacillus formosus* (A6S7) inhibited the growth of MRSA, *K. pneumoniae P. aeruginosa*, *S. aureus,* and *S. typhi* with the zone of inhibition of 24 mm, 22 mm, 22 mm, 21 mm, and 24 mm, respectively. *Bacillus subtilis* (A1S6) inhibited the growth of *E. coli*, *S. typhi*, *K. pneumoniae*, MRSA, and *S. aureus* with the zone of inhibition of 28 mm, 28 mm, 27 mm, 23 mm, and 22 mm, respectively (Figure 1). All the bacterial strains exhibited the best activity against *E. coli*, *S. aureus*, *K. pneumoniae*, *Acinetobacter* *baumannii*, *P. aeruginosa.* In contrast, the least activity was observed against *P. aeruginosa* and MRSA. The MIC of (A1S10) against *S. typhi*, *S. aureus,* and *E. coli* was 0.412, 1.25, and 0.312 mg/mL, respectively. Ethyl acetate extract of bacterial isolates exhibited MIC values of 0.312, 0.422, 1.25 mg/mL, and 2.5 mg/mL against *S. typhi*, *S. aureus,* and *E. coli*, respectively.

### 3.5. Detection of MIC of Crude Bacterial Extracts

**Figure 2:**
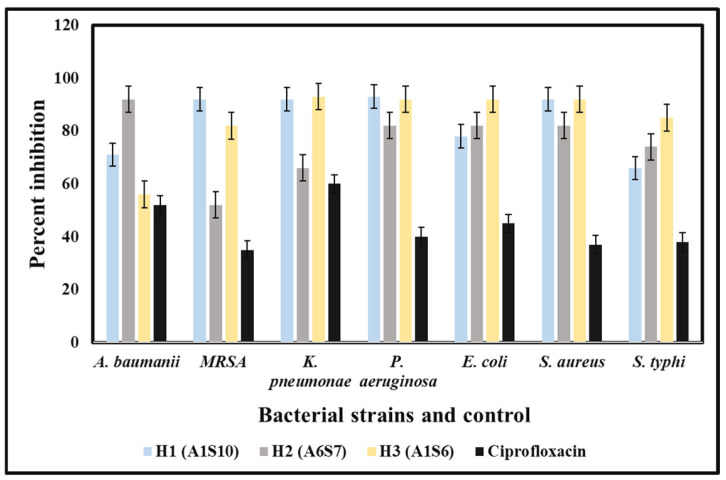
MIC results are shown by crude extracts of bacterial species against MDR strains with variable concentrations.

The bacterial extracts were able to inhibit the growth of tested MDR strains isolated from hospital patients. The least concentration of the methanolic extracts that could stop the growth of pathogenic bacteria was 100 mg/mL for all three bacterial isolates (Figure 2).

### 3.6. Antifungal Potency of Bacterial Extracts

**Figure 3:**
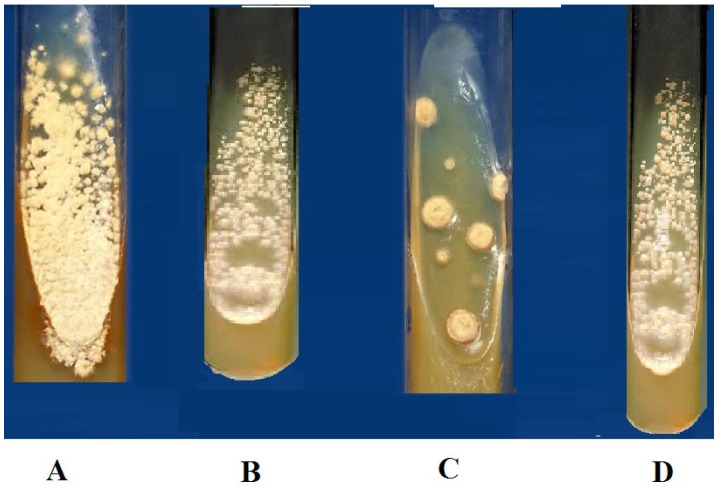
Test tube test for the evaluation of the antifungal activity of metabolites and control (3 mg/mg concentration); (**A**) Control (Amphotericin), (**B**) A_1_S_10_ highest inhibition against *Aspergillus flavus, **(*****C**) A_6_S_7_ the highest inhibition against *Aspergillus niger,* (**D**) A_1_S_6_ highest inhibition against *Aspergillus niger*.

Ethyl acetate extract of bacterial species was analyzed to observe its antifungal potency against the pathogenic strains of fungi using the tube dilution method. Bacterial extracts were tested against two pathogenic fungal species; *Aspergillus flavus* and *Aspergillus niger* (Table 4, Figure 3).

### 3.7. GC-MS Analysis 

The results of GC-MS evaluation indicated the presence of several compounds in crude extracts of bacterial species. The most important and highest components exhibited in the crude extract analyzed with the help of GC-MS are explained in Table 5, Table 6 and Table 7, describing where compounds observed in this study were previously identified. These compounds showed resemblance with the natural products of plant and bacterial origin. The GC–MS data analysis revealed that most were derivatives of volatile compounds such as alkaloids, esters, ethers, and phenolic compounds.

GC-MS analysis revealed propanoic acid, oxalic acid, phenol, and 1,3,5-Trioxaneas the major metabolites present in the extracts of *Bacillus* species. Other compounds viz.9-octadecenoic acid (z)-, 2-hydroxyl-1,3-propanedyl ester, cholestan-3-ol,2methylene (3a,5a), stearic acid, chondrilla sterol, octadeccenoic acid, hexadecanoic acid, cyclobutane, and dasycarpidan were also detected possessing antibacterial, antifungal activity, and antioxidant activities.

### 3.8. Molecular Characterization

**Figure 4:**
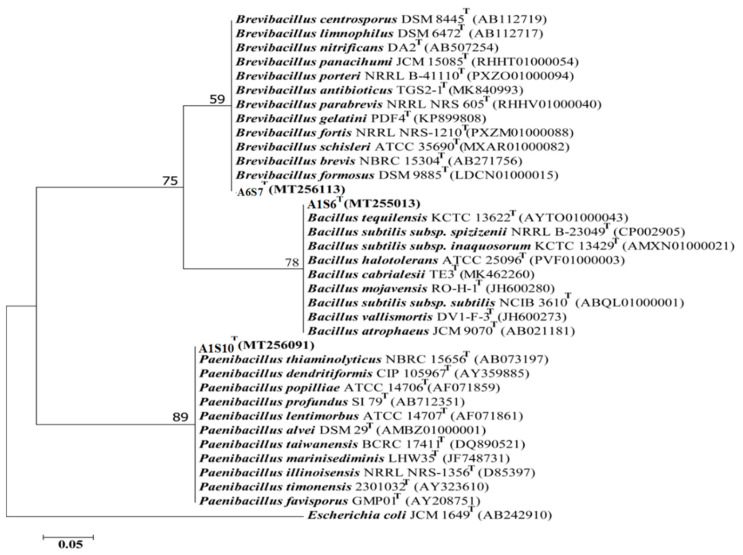
The phylogenetic tree by the Neighbour-joining model was constructed based on 16S rRNA gene sequences representing three different genera, i-e. *Bacillus*, *Brevibacillus* and *Paenibacillus* respectively. The bootstrap value above 60 (1000 replicates) is shown at branch nodes. *E. coli* JCM 1649 (AB242910) was used as an outgroup. Bar, 0.05 substitutions per nucleotide position.

Three (n = 3) bacterial isolates were obtained with increased antimicrobial activities from variable samples. Phylogenetic analysis of the 16S rRNA gene sequences indicated that all the three candidate bacterial isolates, A6S7, A1S10, and A1S6, belong to three different bacterial genera, i.e., *Brevibacillus, Paenibacillus,* and *Bacillus,* respectively (Figure 4) as they cluster with the above-mentioned bacterial *spp*. in the phylogenetic tree.

The top hit sequence similarity determined that these bacterial isolates presumably belong to *Brevibacillus formosus* (99%), *Bacillus Subtilis* (99%), and *Paenibacillus dendritiformis* (99%), respectively (Table 8). The presumably described taxonomy was validated by tree topology and high bootstrap values obtained after phylogenetic analysis.

## 4. Discussion

Soil is a complex and diverse habitat with extensive microbial communities and composition, thus providing highly versatile metabolic biosynthesis and a source of diverse secondary metabolites. Nearly 500 antibiotics are found each year, in which 60% of antibiotics are obtained from soil microbes. Recent analyses have shown that soil screening for antimicrobial activities has been carried out in many parts of the world. A teaspoon of soil contains a hundred million to one billion bacteria active in each acre of the soil. Soil microflora was interesting to investigate, and microbes are considered tiny antimicrobial factories that produce biologically active secondary metabolites. The extreme microbial diversity, abundance and structure also correlate with their diverse metabolic activities, which result in producing countless metabolites with numerous activities, including antimicrobial, anti-parasitic, anti-cancerous and anti-pesticidal activities, etc. The current study aimed to investigate selected soil microbial communities for their potential to produce antimicrobial activities. Twenty different bacterial isolates were found after several steps of isolation, identification on various general purpose, and selected bacterial growth media along with biochemical investigations. In recent years, several microorganisms that can produce antibiotics grown in suitable cultures have increased the probability of developing novel antibiotics to encounter/control untreated infectious diseases. Various studies confirmed soil microflora produce antimicrobial compounds having pharmaceutical and biotechnological applications [21,22].

Three bacterial isolates with antibacterial activity were found positive for fermentation of sugars such as glucose, arabinose, sucrose, starch, rhamnose, glycerol, glycogen, lactose, fructose, raffinose, maltose, and mannitol. All these bacterial isolates were also positive for indole, methyl red, Voges–Proskauer, citrate utilization, catalase, oxidase, cellulose test, triple sugar iron, nitrate reduction, and amino acid utilization tests (Table 1 and Table 2). A study by Tariq, Sudha [23] reported the same results of biochemical analysis and sugar fermentation. Isolated bacterial strains were partially characterized based on different sugars fermentation and biochemical tests. These results showed similarities with previous literature [24].

Our results showed that *Bacillus subtilis*, *Paenibacillus,* and *Brevibacillus* inhibit the growth of multidrug-resistant bacterial strains, which is a contiguous finding previously reported [25,26]. It was also noticed that the crude extract of all three bacterial isolates showed prominent antifungal activities against chosen fungal species (Figure 3 and Table 4). Previous studies determined that the inhibitory effect of *Bacillus* is due to a change in pH in the growth medium or due to the production of volatile compounds. Several other studies reveal that *Bacillus* produces polypeptide antibacterial compounds such as bacitracin, polymyxin, gramicidin S., and tyrothricin. These compounds are effective against a wide range of Gram-positive and Gram-negative bacteria [27].

A previous study revealed that three different bacterial genera were isolated and investigated against MDRS [28]. Another study also found almost similar results [29]. Some of the studies reported that *Brevibacillus* ssp. [25,30] showed robust antimicrobial activity against pathogenic bacteria and fungi. A similar study was conducted on the activity of *Brevibacillus* against MDRS [28]. The GC-MS studies of the bacterial extracts revealed compounds presence (Table 5, Table 6 and Table 7). Mostly these compounds were structurally similar to natural products of plant and bacterial origin and were derivatives of volatile compounds such as esters, alkaloids, ethers and phenolics, etc. The most prominent metabolites of the exact used in this study include propanoic acid, oxalic acid, phenol and 1,3,5-Trioxaneas, possessing antibacterial antifungal activity and antioxidant activities.

Similar observations were reported in several cases; for example, Pecilocin Bb produced by the *Brevibacillus* from soil [26,31] is a bacteriocin-like inhibitory substance (BLIS) that is stable within the pH range of 1.0–9.0, resistant to heat (100 °C for 30 min), as well as detergents and organic solvents. Kim, Bae [32] demonstrated the broad-spectrum antimicrobial activity of *Paneibacillus*. The study also found a variety of secondary metabolites, which were analyzed by GC-MS, including chondrillasterol, stigmasterol, benzedicarboxylic acid, and octadeconoic acid. A similar study was conducted by [33] with the same compounds isolated from plants showing antimicrobial activity against MRSA and *E. coli*. Another similar study was conducted by Phuong, Han [34] on marine *Bacillus subtilis* strain HD16b producing benz dicarboxylic acid and octadecanoic acid.

Molecular investigation revealed the taxonomy of three different isolated belonging to three different genera, including *Bacillus*, *Paenibacillus*, and other *Brevibacillus.* Based on phylogenetic analysis and top hit sequence similarity results, and supported by a high bootstrap value, it was provisionally assumed that the three most active candidates of bacterial isolates A6S7, A1S10, and A1S6 belong to *Brevibacillus formosus* (99%), *Bacillus subtilis* (99%), and *Paenibacillus dendritiformis* (99%), respectively. It was reported by Amin, Rakhisi [35] that the *Bacillus spp*. is very common in soil. For their successful colonization in various environmental habitats, their highly resistant endospores help them. On the other hand, the isolation strategy applied favors the spore-formers. Identification of three different bacterial strains and their anti microbial activity would help to facilitate microbial screening in the future and the isolation of active metabolites against MDRS.

## 5. Conclusions

In the current study, soil bacterial isolates identified as three different genera capable of inhibiting the growth of multidrug-resistant bacterial strains have been found. Crude extracts from three isolated bacterial strains were potent against bacterial and fungal strains when tested by well-diffusion and MIC methods. The GC-MS analysis identified several volatile inhibitory compounds, including esters, phenolics, ethers, and alkaloids, possibly contributing to antimicrobial activity. In light of GCMS results, it was concluded that bacterial extract possessed potential compounds used as antimicrobial agents against various MDRS. It is expected that a detailed study of a similar nature could further explore novel microbial candidates with unexplored compounds/metabolites having significant antimicrobial potential. Thus, it could be a possible way to reduce the burden and threat of MDRS.

## Figures and Tables

**Table 1:** Colony morphology and microscopic presentation of isolated bacterial species.

Bacterial Species	Media	Colony Color and Texture	Microscopic Presentation
*Brevibacillus formosus*	*Bacillus* Medium.	White, smooth texture	Gram positive, spore-forming, Rod.
*Bacillus Subtilis*	*Bacillus* Medium.	White, irregular, flat	Gram positive, spore-forming, Rod
*Paenibacillus dendritiformis*	*Bacillus* Medium (ATCC Medium 552).	Pink, rough texture	Gram positive, spore-forming, Rod.

**Table 2:** Biochemical Characterization of bacterial isolates from Agricultural, Marshy, and Forest soil collected from Khyber Pakhtunkhwa, Pakistan.

Biochemical Test	Agriculture Soil	Marshy Soil	Forest Soil
Indole Test	+	-	-
Methyl Red Test	+	+	+
Voges Proskauer	+	+	+
Citrate Test	+	+	+
Triple Sugar Iron Agar Test	K/A	K/A	K/A
Catalase test	+	+	+
Oxidase test	+	+	+
Casein Hydrolysis	+	+	+
Gelatin Hydrolysis	+	+	+
Urease Test	+	+	+
Cellulose Test	+	+	+
Nitrate Reduction test	+	+	+
Starch Hydrolysis	+	+	+
**Identified as;**	***Brevibacillus***	***Bacillus subtilis***	***Paenibacillus***

**Table 3:** Different Carbohydrate fermentation of bacterial Isolates from Agricultural, Marshy, and Forest soil.

Carbohydrate Fermentation	Agriculture Soil	Marshy Soil	Forest Soil
Glucose	+	+	+
Lactose	+	+	+
Maltose	+	+	+
Mannitol	+	+	+
Sucrose	+	+	+
Arbinose	+	-	-
Starch	+	+	+
Dextrose	+	+	+
Glycogen	+	+	+
Galactose	+	+	+
Fructose	+	+	+
Raffinose	+	-	+
Rhamnose	-	-	-
Glycerol	+	+	+
Oxidative Fermentation test	+	+	+
**Identified as;**	***Brevibacillus***	***Bacillus subtilis***	***Paenibacillus***

**Table 4:** Antifungal activity of bacterial extracts (3 mg/mL) against pathogenic fungal species.

S. No.	Bacterial Extract (3 mg/mL)	Antifungal Activity of Bacterial Extracts in Percentage	
		*Aspergillus flavus*	*Aspergillus niger*
1	H_1_(A_1_S_10_)	63.5%	56.5%
2	H_2_(A_6_S_7_)	53%	77.25%
3	H_3_(A_1_S_6_)	68.8%	87.7%
4	Amphotericin	25%	27.3%

**Table 5:** Major constituents of bacterial extract H1 (A1S10) were identified by GC–MS analysis.

Compound Name	Molecular Formula	Molecular Mass	Retention Time (min)	CAS#	Library	Probability	% of Area
9-Octadecenoic acid (z)-, 2-hydroxyl-1,3-propanedyl ester	C_39_H_72_O_5_	620 g/mol	1.98	2465-32-9	MAIN LIB	6.38	0.08
Oxalic Acid	C_2_H_2_O_4_	90 g/mol	0.67	144-62-7	Mist-msms	18.77	71.11
Naphthalene, 1,2,3,4-tetrahydro-66-methyl	C_11_H_14_	146 g/mol	7.78	1680-51-9	MAIN LIB	27.71	0.88
Cholestan-3-ol, 2-methylene (3a, 5a)	C_28_H_48_O	400 g/mol	400		MAIN LIB	12.09	0.39
Stearic acid	C_39_H_78_O_3_	594 g/mol	19.42	17367-40-7	MAIN LIB	10.69	0.02
Propanoic Acid	C_8_H_16_O_2_	144 g/mol	0.67	97-87-0	Rep Lib	18.04	71.11
Chondrilla sterol	C_29_H_48_O	412 g/mol	20.88	481-17-4	Main lib	19.83	0.24
Stegmasterol	C_29_H_48_O	412 g/mol	20.88	83-48-7	Rep Lib	13.20	0.24
Trifluoroacetic acid	C_20_H_37_F_3_O_2_	366 g/mol	12.02	79392-43-1	Main Lib	4.99	0.31
Benzenedicaroxylic acid, diisoocyl ester	C_24_H_38_O_4_	390 g/mol	16.48	27554-26-3	Main Lib	41.23	0.16

**Table 6:** Major constituents of bacterial extract of H2 (A6S7) were identified by GC–MS analysis.

Compound Name	Molecular Formula	Molecular Mass	Retention Time (min)	CAS#	Library	Probability	% of Area
Phenol	C_6_H_6_O	94 g/mol	4.65	108-95-2	Rep Lib	67.81	13.52
Octadeccenoic acid	C_57_H_10_	884 g/mol	3.42	537-39-3	Main Lib	41.23	0.16
Aspidospermidin	C_23_H_30_N_2_O_5_	414 g/mol	9.49	2122-26-1	Main Lib	13.09	0.19
Hexadecanoic acid	C_16_H_32_O_2_	256 g/mol	13.3	57-10-3	Main lib	21.83	0.99
Steric acid	C_39_H_78_O_3_	594 g/mol	17.66	17367-40-7	Main lib	10.79	0.13

**Table 7:** Major constituents of bacterial extract H3(A1S6) were identified by GC–MS analysis.

Compound Name	Molecular Formula	Molecular Mass	Retention Time (min)	CAS#	Library	Probability	% of Area
Cyclobutane	C_7_H_8_	92 g/mol	1.33				
1,3,5-Trioxane	C_3_H_6_O_3_	90 g/mol	0.71	110-88-3	Main lib	83.68	61.30
Phenol	C_14_H_22_O	206 g/mol	9096	96-76-4	Main lib	51.04	1.87
Dasycarpidan	C_20_H_26_N_2_O_2_	326 g/mpl	19.41				

**Table 8:** Identification of bacterial species based on sequences similarities.

S. No.	Isolates	Source	16S r RNA Amplified Region Length	% Similarity	NCBI Accession No
1.	**A6S7**	Agricultural Soil	635 base pair	99% with *Brevibacillus formosus*	MT256113
2.	**A1S6**	Marshy Soil	848 base pair	99% with *Bacillus Subtilis*	MT255013
3.	**A1S10**	Forest Soil	645 base pair	99% with *Paenibacillus* *dendritiformis*	MT256091

## Data Availability

The authors confirm that the data supporting the findings of this study are available within the article.
